# Dimeth­yl(4-methyl­phen­yl)ammonium naphthalene-1,5-disulfonate dihydrate

**DOI:** 10.1107/S1600536811037068

**Published:** 2011-09-17

**Authors:** Bin Wei

**Affiliations:** a Ordered Matter Science Research Center, Southeast University, Nanjing 211189, People’s Republic of China

## Abstract

The asymmetric unit of the organic–inorganic hybrid salt, 2C_9_H_14_N^+^·C_10_H_6_O_6_S_2_^2−^·2H_2_O, consists of one dimeth­yl(4-methyl­phen­yl)ammonium cation, one half of a naphthalene-1,5-disulfonate anion lying on a crystallographic centre of inversion, and one water mol­ecule. In the crystal, O—H⋯O(S) and N—H⋯OH_2_ hydrogen bonds link the cations and anions forming ring motifs.

## Related literature

The title compound was obtained during attempts to obtain dielectric-ferroelectric materials. For general background to ferroelectric metal-organic frameworks, see: Wu *et al.* (2011 ▶); Fu *et al.* (2009 ▶); Ye *et al.* (2006 ▶); Zhang *et al.* (2008 ▶); Zhang *et al.* (2010 ▶). 
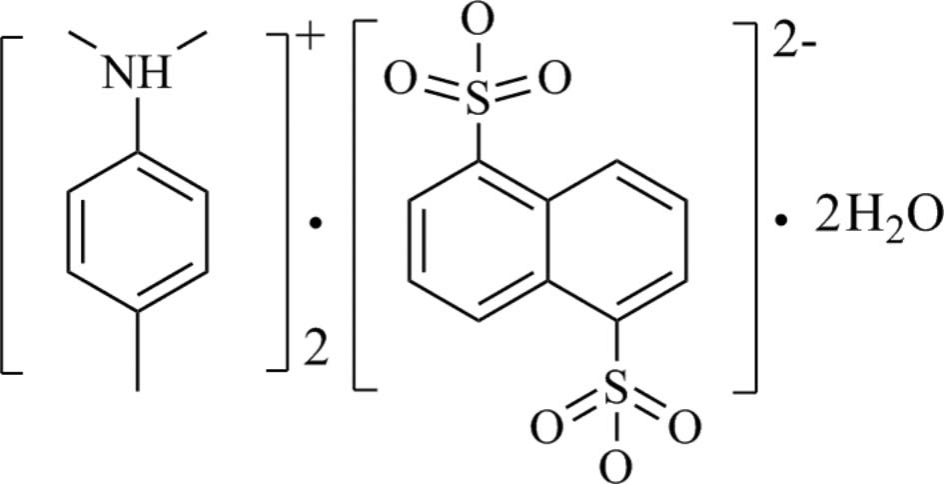


## Experimental

### 

#### Crystal data

2C_9_H_14_N^+^·C_10_H_6_O_6_S_2_^2−^·2H_2_O
*M* *_r_* = 594.74
Triclinic, 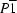

*a* = 9.2660 (19) Å
*b* = 9.882 (2) Å
*c* = 10.260 (2) Å
α = 109.59 (3)°
β = 115.79 (3)°
γ = 98.39 (3)°
*V* = 748.3 (3) Å^3^
*Z* = 1
Mo *K*α radiation
μ = 0.23 mm^−1^
*T* = 293 K
0.20 × 0.20 × 0.20 mm

#### Data collection

Rigaku SCXmini diffractometer
Absorption correction: multi-scan (*CrystalClear*; Rigaku, 2005 ▶) *T*_min_ = 0.955, *T*_max_ = 0.955
7762 measured reflections
3421 independent reflections
2951 reflections with *I* > 2σ(*I*)
*R*_int_ = 0.026

#### Refinement

*R*[*F* ^2^ > 2σ(*F* ^2^)] = 0.040
*wR*(*F* ^2^) = 0.115
*S* = 1.06
3421 reflections
197 parameters
H atoms treated by a mixture of independent and constrained refinement
Δρ_max_ = 0.28 e Å^−3^
Δρ_min_ = −0.34 e Å^−3^

### 

Data collection: *CrystalClear* (Rigaku, 2005 ▶); cell refinement: *CrystalClear*; data reduction: *CrystalClear*; program(s) used to solve structure: *SHELXS97* (Sheldrick, 2008 ▶); program(s) used to refine structure: *SHELXL97* (Sheldrick, 2008 ▶); molecular graphics: *SHELXTL* (Sheldrick, 2008 ▶); software used to prepare material for publication: *SHELXTL*.

## Supplementary Material

Crystal structure: contains datablock(s) I, global. DOI: 10.1107/S1600536811037068/jh2329sup1.cif

Structure factors: contains datablock(s) I. DOI: 10.1107/S1600536811037068/jh2329Isup2.hkl

Supplementary material file. DOI: 10.1107/S1600536811037068/jh2329Isup3.cml

Additional supplementary materials:  crystallographic information; 3D view; checkCIF report

## Figures and Tables

**Table 1:** Hydrogen-bond geometry (Å, °)

*D*—H⋯*A*	*D*—H	H⋯*A*	*D*⋯*A*	*D*—H⋯*A*
O4—H4*B*⋯O3^i^	0.85 (3)	1.93 (3)	2.778 (3)	176 (3)
O4—H4*B*⋯S1^i^	0.85 (3)	2.96 (3)	3.753 (3)	157 (2)
N1—H10⋯O4^ii^	0.89 (2)	1.84 (2)	2.723 (2)	174.2 (19)
O4—H4*A*⋯O1^iii^	0.84 (3)	2.01 (3)	2.846 (2)	171 (2)
O4—H4*A*⋯S1^iii^	0.84 (3)	2.87 (3)	3.6569 (18)	156 (2)

Symmetry codes: (i) 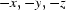; (ii) 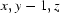; (iii) 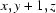.

**Fig. 1.**
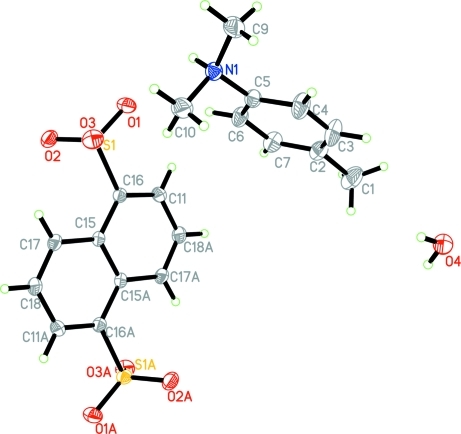
The molecular structure of the title compound, with the atomic numbering scheme. Displacement ellipsoids are drawn at the 30% probability level.

**Fig. 2.**
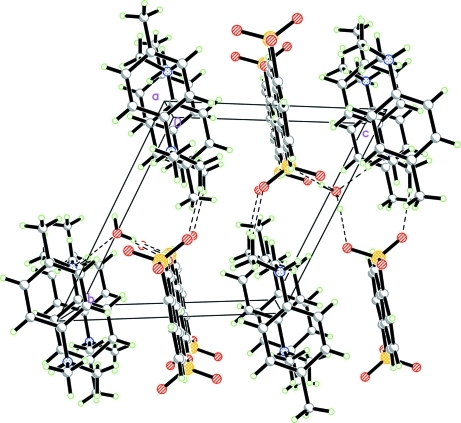
A view of the packing of the title compound, stacking along the a axis. Dashed lines indicate hydrogen bonds.

## supplementary crystallographic information

### Comment

Dielectric-ferroelectric constitute an interesting class of materials, comprising organic ligands, metal-organic coordination compounds and organic-inorganic hybrids(Fu *et al.*, 2009; Zhang *et al.*, 2010; Zhang *et al.*, 2008; Ye *et al.*, 2006). Unfortunately,the dielectric constant of the title compound as a function of temperature indicates that the permittivity is basically temperature-independent, below the melting point (385k-387k) of the compound, we have found that cyclohexylammonium 4-methoxy-benzoate has no dielectric disuniform from 80 K to 405 K. Herein we descibe the crystal structure of this compound.

Regarding its crystal structure,the asymmetric unit of the title compound consists of a dimethyl(4-methylphenyl)ammonium cation, a half of naphthalene-1,5-disulfonate anion and a water molecule(Fig. 1). The free water molecules connected cations and anions by intermolecular hydrogen bonds involving O—H···S, O—H···O and N—H···O which makes great contribution to the stability of the crystal structure,and these hydrogen bonds link the cations, water molecules and anions into a chains along the *c* axis(Fig. 2 and Tab. 1).

### Experimental

The title compound was obtained by the addition of naphthalene-1,5-disulfonate acid (3.62 g, 0.01 mol) to a solution of dimethyl(4-methylphenyl)amine (2.72 g, 0.02 mol) in water, in the stoichiometric ratio 1: 2. Good quality single crystals were obtained by slow evaporation after two days(the chemical yield is 35%).

### Refinement

All H atoms were placed in geometrically idealized positions and constrained to ride on their parent atoms with C—H = 0.96 Å, O—H = 0.84 to 0.85 Å, N—H = 0.89 Å and with *U*_iso_(H) = 1.2 *U*_iso_(C, O) or 1.5 *U*_iso_(C) for methyl H atoms.

### Crystal data

**Table:**

2C_9_H_14_N^+^·C_10_H_6_O_6_S_2_^2^^−^·2H_2_O	*Z* = 1
*M**_r_* = 594.74	*F*(000) = 314
Triclinic, *P*1	*D*_x_ = 1.315 Mg m^−^^3^
*a* = 9.2660 (19) Å	Mo *K*α radiation, λ = 0.71073 Å
*b* = 9.882 (2) Å	θ = 3.0–27.5°
*c* = 10.260 (2) Å	µ = 0.23 mm^−^^1^
α = 109.59 (3)°	*T* = 293 K
β = 115.79 (3)°	Block, colorless
γ = 98.39 (3)°	0.20 × 0.20 × 0.20 mm
*V* = 748.3 (3) Å^3^	

### Data collection

**Table:**

Rigaku SCXmini diffractometer	3421 independent reflections
Radiation source: fine-focus sealed tube	2951 reflections with *I* > 2σ(*I*)
graphite	*R*_int_ = 0.026
CCD_Profile_fitting scans	θ_max_ = 27.5°, θ_min_ = 3.3°
Absorption correction: multi-scan (*CrystalClear*; Rigaku, 2005)	*h* = −12→12
*T*_min_ = 0.955, *T*_max_ = 0.955	*k* = −12→12
7762 measured reflections	*l* = −13→13

### Refinement

**Table:**

Refinement on *F*^2^	Secondary atom site location: difference Fourier map
Least-squares matrix: full	Hydrogen site location: inferred from neighbouring sites
*R*[*F*^2^ > 2σ(*F*^2^)] = 0.040	H atoms treated by a mixture of independent and constrained refinement
*wR*(*F*^2^) = 0.115	*w* = 1/[σ^2^(*F*_o_^2^) + (0.0587*P*)^2^ + 0.1813*P*] where *P* = (*F*_o_^2^ + 2*F*_c_^2^)/3
*S* = 1.06	(Δ/σ)_max_ = 0.015
3421 reflections	Δρ_max_ = 0.28 e Å^−^^3^
197 parameters	Δρ_min_ = −0.34 e Å^−^^3^
0 restraints	Extinction correction: *SHELXL97* (Sheldrick, 2008), Fc^*^=kFc[1+0.001xFc^2^λ^3^/sin(2θ)]^-1/4^
Primary atom site location: structure-invariant direct methods	Extinction coefficient: 0.030 (5)

### Special details

**Table:**

Geometry. All e.s.d.'s (except the e.s.d. in the dihedral angle between two l.s. planes) are estimated using the full covariance matrix. The cell e.s.d.'s are taken into account individually in the estimation of e.s.d.'s in distances, angles and torsion angles; correlations between e.s.d.'s in cell parameters are only used when they are defined by crystal symmetry. An approximate (isotropic) treatment of cell e.s.d.'s is used for estimating e.s.d.'s involving l.s. planes.
Refinement. Refinement of *F*^2^ against ALL reflections. The weighted *R*-factor *wR* and goodness of fit *S* are based on *F*^2^, conventional *R*-factors *R* are based on *F*, with *F* set to zero for negative *F*^2^. The threshold expression of *F*^2^ > σ(*F*^2^) is used only for calculating *R*-factors(gt) *etc*. and is not relevant to the choice of reflections for refinement. *R*-factors based on *F*^2^ are statistically about twice as large as those based on *F*, and *R*- factors based on ALL data will be even larger.

### Fractional atomic coordinates and isotropic or equivalent isotropic displacement parameters (Å^2^)

**Table:**

	*x*	*y*	*z*	*U*_iso_*/*U*_eq_	
C1	0.5316 (3)	0.4244 (2)	0.3073 (3)	0.0732 (7)	
H1A	0.5825	0.4690	0.2607	0.110*	
H1B	0.6194	0.4366	0.4096	0.110*	
H1C	0.4552	0.4746	0.3232	0.110*	
N1	0.14101 (18)	−0.21478 (16)	−0.12073 (17)	0.0374 (3)	
O1	0.29693 (16)	−0.24956 (14)	0.32982 (15)	0.0444 (3)	
S1	0.13241 (5)	−0.27559 (4)	0.31891 (4)	0.03323 (14)	
C2	0.4338 (2)	0.2567 (2)	0.1950 (2)	0.0487 (4)	
O2	0.10384 (18)	−0.37468 (13)	0.38791 (16)	0.0482 (3)	
C3	0.3514 (3)	0.2034 (2)	0.0300 (3)	0.0649 (6)	
H3	0.3587	0.2722	−0.0123	0.078*	
O3	−0.00765 (16)	−0.32258 (14)	0.15568 (14)	0.0466 (3)	
C4	0.2579 (3)	0.0502 (2)	−0.0752 (2)	0.0590 (6)	
H4	0.2035	0.0167	−0.1865	0.071*	
O4	0.26401 (18)	0.60704 (17)	0.02223 (18)	0.0468 (3)	
C5	0.2464 (2)	−0.05151 (19)	−0.0131 (2)	0.0369 (4)	
C6	0.3283 (2)	−0.0021 (2)	0.1516 (2)	0.0442 (4)	
H6	0.3209	−0.0714	0.1933	0.053*	
C7	0.4220 (3)	0.1516 (2)	0.2548 (2)	0.0495 (5)	
H7	0.4779	0.1848	0.3661	0.059*	
C9	0.1372 (3)	−0.2773 (2)	−0.2766 (2)	0.0580 (5)	
H9A	0.0704	−0.2369	−0.3454	0.087*	
H9B	0.0872	−0.3868	−0.3291	0.087*	
H9C	0.2515	−0.2485	−0.2556	0.087*	
C10	−0.0362 (3)	−0.2446 (2)	−0.1518 (3)	0.0573 (5)	
H10A	−0.0325	−0.2135	−0.0508	0.086*	
H10B	−0.1006	−0.3519	−0.2186	0.086*	
H10C	−0.0897	−0.1877	−0.2060	0.086*	
C11	0.2843 (2)	0.02932 (18)	0.5176 (2)	0.0371 (4)	
H11	0.3775	0.0157	0.5088	0.045*	
C15	−0.00608 (18)	−0.07490 (15)	0.45203 (17)	0.0275 (3)	
C16	0.13837 (19)	−0.09301 (16)	0.43905 (17)	0.0294 (3)	
C17	−0.1605 (2)	−0.19878 (17)	0.37400 (19)	0.0350 (3)	
H17	−0.1695	−0.2968	0.3124	0.042*	
C18	−0.2949 (2)	−0.17579 (18)	0.3883 (2)	0.0412 (4)	
H18	−0.3954	−0.2581	0.3351	0.049*	
H10	0.186 (3)	−0.267 (2)	−0.068 (2)	0.043 (5)*	
H4A	0.268 (3)	0.640 (3)	0.111 (4)	0.073 (8)*	
H4B	0.187 (3)	0.519 (3)	−0.036 (3)	0.075 (8)*	

### Atomic displacement parameters (Å^2^)

**Table:**

	*U*^11^	*U*^22^	*U*^33^	*U*^12^	*U*^13^	*U*^23^
C1	0.0718 (15)	0.0389 (11)	0.0853 (17)	0.0116 (10)	0.0304 (13)	0.0197 (11)
N1	0.0403 (8)	0.0372 (7)	0.0359 (7)	0.0127 (6)	0.0197 (6)	0.0179 (6)
O1	0.0425 (7)	0.0480 (7)	0.0438 (7)	0.0180 (5)	0.0269 (6)	0.0144 (6)
S1	0.0390 (2)	0.0300 (2)	0.0306 (2)	0.01212 (16)	0.01956 (17)	0.01138 (16)
C2	0.0452 (10)	0.0376 (9)	0.0546 (11)	0.0129 (8)	0.0207 (9)	0.0190 (8)
O2	0.0708 (9)	0.0355 (6)	0.0547 (8)	0.0226 (6)	0.0408 (7)	0.0247 (6)
C3	0.0811 (16)	0.0455 (11)	0.0614 (13)	0.0120 (10)	0.0263 (12)	0.0354 (10)
O3	0.0474 (7)	0.0445 (7)	0.0310 (6)	0.0119 (5)	0.0147 (5)	0.0084 (5)
C4	0.0767 (14)	0.0477 (11)	0.0410 (10)	0.0108 (10)	0.0198 (10)	0.0266 (9)
O4	0.0493 (8)	0.0446 (8)	0.0410 (7)	0.0129 (6)	0.0218 (6)	0.0170 (6)
C5	0.0387 (8)	0.0366 (8)	0.0355 (8)	0.0126 (7)	0.0177 (7)	0.0186 (7)
C6	0.0547 (11)	0.0397 (9)	0.0390 (9)	0.0147 (8)	0.0212 (8)	0.0231 (8)
C7	0.0532 (11)	0.0429 (10)	0.0383 (9)	0.0118 (8)	0.0155 (8)	0.0163 (8)
C9	0.0728 (14)	0.0515 (11)	0.0478 (11)	0.0116 (10)	0.0390 (11)	0.0138 (9)
C10	0.0454 (11)	0.0502 (11)	0.0707 (14)	0.0124 (9)	0.0332 (10)	0.0187 (10)
C11	0.0299 (8)	0.0354 (8)	0.0457 (9)	0.0086 (6)	0.0218 (7)	0.0159 (7)
C15	0.0293 (7)	0.0249 (7)	0.0263 (7)	0.0061 (5)	0.0135 (6)	0.0120 (6)
C16	0.0325 (7)	0.0273 (7)	0.0286 (7)	0.0095 (6)	0.0161 (6)	0.0127 (6)
C17	0.0341 (8)	0.0242 (7)	0.0382 (8)	0.0047 (6)	0.0168 (7)	0.0099 (6)
C18	0.0314 (8)	0.0292 (8)	0.0512 (10)	0.0013 (6)	0.0193 (7)	0.0121 (7)

### Geometric parameters (Å, °)

**Table:**

C1—C2	1.506 (3)	C5—C6	1.376 (2)
C1—H1A	0.9600	C6—C7	1.385 (3)
C1—H1B	0.9600	C6—H6	0.9300
C1—H1C	0.9600	C7—H7	0.9300
N1—C5	1.478 (2)	C9—H9A	0.9600
N1—C9	1.490 (2)	C9—H9B	0.9600
N1—C10	1.492 (2)	C9—H9C	0.9600
N1—H10	0.89 (2)	C10—H10A	0.9600
O1—S1	1.4562 (13)	C10—H10B	0.9600
S1—O2	1.4430 (13)	C10—H10C	0.9600
S1—O3	1.4548 (15)	C11—C16	1.364 (2)
S1—C16	1.7883 (16)	C11—C18^i^	1.406 (2)
C2—C3	1.374 (3)	C11—H11	0.9300
C2—C7	1.386 (3)	C15—C17	1.422 (2)
C3—C4	1.384 (3)	C15—C16	1.432 (2)
C3—H3	0.9299	C15—C15^i^	1.432 (3)
C4—C5	1.373 (2)	C17—C18	1.358 (2)
C4—H4	0.9300	C17—H17	0.9300
O4—H4A	0.84 (3)	C18—C11^i^	1.406 (2)
O4—H4B	0.85 (3)	C18—H18	0.9300
			
C2—C1—H1A	109.5	C5—C6—H6	120.3
C2—C1—H1B	109.5	C7—C6—H6	120.3
H1A—C1—H1B	109.5	C6—C7—C2	121.13 (17)
C2—C1—H1C	109.5	C6—C7—H7	119.4
H1A—C1—H1C	109.5	C2—C7—H7	119.4
H1B—C1—H1C	109.5	N1—C9—H9A	109.5
C5—N1—C9	114.42 (14)	N1—C9—H9B	109.5
C5—N1—C10	111.22 (14)	H9A—C9—H9B	109.5
C9—N1—C10	110.28 (16)	N1—C9—H9C	109.5
C5—N1—H10	107.1 (13)	H9A—C9—H9C	109.5
C9—N1—H10	107.0 (13)	H9B—C9—H9C	109.5
C10—N1—H10	106.4 (13)	N1—C10—H10A	109.5
O2—S1—O3	113.10 (9)	N1—C10—H10B	109.5
O2—S1—O1	113.24 (8)	H10A—C10—H10B	109.5
O3—S1—O1	112.12 (8)	N1—C10—H10C	109.5
O2—S1—C16	106.42 (7)	H10A—C10—H10C	109.5
O3—S1—C16	105.32 (8)	H10B—C10—H10C	109.5
O1—S1—C16	105.85 (8)	C16—C11—C18^i^	120.47 (15)
C3—C2—C7	117.88 (17)	C16—C11—H11	119.8
C3—C2—C1	121.06 (19)	C18^i^—C11—H11	119.8
C7—C2—C1	121.1 (2)	C17—C15—C16	122.94 (13)
C2—C3—C4	121.90 (18)	C17—C15—C15^i^	118.74 (17)
C2—C3—H3	119.0	C16—C15—C15^i^	118.32 (16)
C4—C3—H3	119.1	C11—C16—C15	120.69 (14)
C5—C4—C3	119.14 (18)	C11—C16—S1	118.17 (12)
C5—C4—H4	120.4	C15—C16—S1	121.14 (11)
C3—C4—H4	120.4	C18—C17—C15	120.86 (15)
H4A—O4—H4B	105 (2)	C18—C17—H17	119.6
C4—C5—C6	120.44 (17)	C15—C17—H17	119.6
C4—C5—N1	121.04 (15)	C17—C18—C11^i^	120.91 (15)
C6—C5—N1	118.48 (15)	C17—C18—H18	119.5
C5—C6—C7	119.50 (16)	C11^i^—C18—H18	119.5

Symmetry codes: (i) −*x*, −*y*, −*z*+1.

### Hydrogen-bond geometry (Å, °)

**Table:**

*D*—H···*A*	*D*—H	H···*A*	*D*···*A*	*D*—H···*A*
O4—H4B···O3^ii^	0.85 (3)	1.93 (3)	2.778 (3)	176 (3)
O4—H4B···S1^ii^	0.85 (3)	2.96 (3)	3.753 (3)	157 (2)
N1—H10···O4^iii^	0.89 (2)	1.84 (2)	2.723 (2)	174.2 (19)
O4—H4A···O1^iv^	0.84 (3)	2.01 (3)	2.846 (2)	171 (2)
O4—H4A···S1^iv^	0.84 (3)	2.87 (3)	3.6569 (18)	156 (2)

Symmetry codes: (ii) −*x*, −*y*, −*z*; (iii) *x*, *y*−1, *z*; (iv) *x*, *y*+1, *z*.
